# Pain Management in Animals with Oncological Disease: Opioids as Influencers of Immune and Tumor Cellular Balance

**DOI:** 10.3390/cancers16173015

**Published:** 2024-08-29

**Authors:** Ana Vidal Pinheiro, Gonçalo N. Petrucci, Amândio Dourado, Filipe Silva, Isabel Pires

**Affiliations:** 1 Animal and Veterinary Research Centre (CECAV), University of Trás-os-Montes and Alto Douro, 5000-801 Vila Real, Portugal; goncalopetrucci@gmail.com (G.N.P.); fsilva@utad.pt (F.S.); ipires@utad.pt (I.P.); 2 Department of Veterinary Sciences, University of Trás-os-Montes and Alto Douro (UTAD), 5000-801 Vila Real, Portugal; amandio.dourado@onevetgroup.pt; 3 Animal and Veterinary Department, University Institute of Health Sciences, Advanced Polytechnic and University Cooperative, CRL, 4585-116 Gandra, Portugal; 4 School of Agrarian Sciences, Polytechnic Institute of Viana do Castelo, Refoidos do Lima, 4990-706 Ponte de Lima, Portugal; 5 Onevetgroup Hospital Veterinário do Porto (HVP), 4250-475 Porto, Portugal; 6 Center for Investigation Vasco da Gama (CIVG), Department of Veterinary Sciences, Vasco da Gama University School (EUVG), 3020-210 Coimbra, Portugal

**Keywords:** cancer pain, opioids, immune effects, tumor cells, morphine, tramadol, methadone, fentanyl, buprenorphine, butorphanol

## Abstract

**Simple Summary:**

Advances in pain research challenge the concept that animals lack pain senses, showing that they have similar neural pathways to humans and experience pain similarly. Understanding brain circuits for effective pain control is crucial for adjusting pain control to individual patient responses and conditions. Pain management in oncological patients aims to lessen the impact of tumor cell development and its consequences on the immune system. Researchers have focused on improving algological approaches to better respond to patient needs, which requires a deeper understanding of how analgesics work, interact with other drugs, and affect patients’ conditions. Opioids, although linked to tumor progression, remain the mainstay for managing oncologic pain.

**Abstract:**

Advancements in understanding pain physiopathology have historically challenged animals’ absence of pain senses. Studies have demonstrated that animals have comparable neural pain pathways, suggesting that cats and dogs likely experience pain similarly to humans. Understanding brain circuits for effective pain control has been crucial to adjusting pain management to the patient’s individual responses and current condition. The refinement of analgesic strategies is necessary to better cater to the patient’s demands. Cancer pain management searches to ascertain analgesic protocols that enhance patient well-being by minimizing or abolishing pain and reducing its impact on the immune system and cancer cells. Due to their ability to reduce nerve sensitivity, opioids are the mainstay for managing moderate and severe acute pain; however, despite their association with tumor progression, specific opioid agents have immune-protective properties and are considered safe alternatives to analgesia for cancer patients.

## 1. Introduction

Pain generally has a negative physiological and behavioral impact on animals [1]. The neurobiological mechanisms of pain induce metabolic, autonomic, and behavioral changes, affecting animals’ welfare and quality of life [1]. Algology focuses on improving pain management in animals undergoing acute and chronic painful processes [2,3,4].

In oncologic patients undergoing surgery, the main risk of tumor dissemination occurs in the perioperative period [5,6], during which stress [7], pain [8], anesthetic and analgesic agents [9,10], and surgical procedures influence the immune and tumor cellular balance [11,12]. The mechanism that has been suggested for dissemination is the possible depression of both the immune and the sympathetic nervous system following surgical stress [12].

Opioid agents remain the basis of moderate and severe pain management [8,13,14,15] as well as oncologic pain [4,8,16] due to their effective capacity to block nerve sensibility [17]. Even though there is no consensus among the veterinary medical community and the most recent guidelines [18], more than 80% of veterinarians report using opioids for chronic pain management often or always, highlighting the widespread acceptance of opioids in pain control across different medical fields [19]. Furthermore, human studies emphasize the knowledge and attitudes of oncology nurses regarding cancer pain management, reinforcing the critical role of opioids in this context [20]. On the other hand, even though opioids offer improved benefits for patient welfare through great pain-feeling control, they could, at the same time, potentially induce unfavorable effects on the immune system [8] and promote tumor cell growth [21,22]. Multimodal pain management, which involves combining multiple analgesic drug classes or techniques, targets different points along the pain pathway, enhancing pain control by decreasing the adverse effects of opioids and avoiding sensitization phenomena [23,24]. Likewise, anesthesia with a low dose of opioids or even opioid-free analgesia could prevent or minimize opioid-related side effects [25,26] while simultaneously increasing analgesic efficacy [27,28]. However, some authors suggest that specific opioid agents could be an exception, as they have demonstrated immune-protective properties and are considered safe analgesic alternatives in oncologic patients [18].

Even though there have been improvements in analgesic strategies, opioids are still required for pain control in major oncologic diseases. This leads to more research and knowledge of these analgesics’ pro-immune and anti-tumor properties, preserving immune function and minimizing the risk of tumor development [19].

The objective of this review was to analyze the mechanisms of the nociceptive process of pain, the effect of opioid use on the immune system, and their influence on tumor growth mechanisms in oncological patients.

## 2. Search Methodology

We conducted a comprehensive literature search initially on digital databases ResearchGate and PubMed/Medline using the following terms: “pain disorders”; “oncological pain”; “anaesthetic strategies in oncological surgery”; “analgesia and cancer”; “analgesia and cancer recurrence and metastasis”; “veterinary anaesthesia oncology”; “anaesthesia and cancer recurrence and metastasis in dogs”; “opioids and immune system”; “opioids and cancer effects”; and “anaesthesia opioids-free”. The search terms were used for finding full-text, randomized controlled trials, clinical trials, and meta-analyses.

Given the diversity of the studies, the small sample sizes, the lack of veterinary studies, and the specific nature of the research question, a narrative review was deemed most suitable. We reviewed titles, abstracts, full-text articles, and references from these articles to provide a comprehensive overview of the current evidence on pain mechanisms, their impact on animal welfare, and the various strategies for pain management in oncologic patients. Emphasis was placed on the effectiveness of multimodal analgesia and adjunctive therapies in improving pain control while minimizing the adverse effects of opioids.

## 3. Mechanisms of Pain and Its Important Role in Animal Welfare

Pain has long been acknowledged in clinical medical practice for its detrimental impact on several organ systems [29]. Feeling pain can induce the body’s stress and promote several metabolic and endocrine disorders, influencing patient recovery processes [29,30]. In oncologic diseases, pain can also negatively affect the immune system, decreasing the number and activity of immune cells and fostering a favorable environment for tumor growth and spread [8].

It was once believed that animals were incapable of experiencing pain or that their perception of pain differed from that of humans [31]. Moreover, it was also thought that animals’ pain following injuries could be advantageous, restricting their mobility and thereby averting subsequent possible damages [31]. However, advancements in understanding the mechanisms underlying pain have led to a shift in this perspective, showing that animals share similar neural circuits in pain generation, conduction, and regulation with humans [31]. According to the International Association for the Study of Pain, pain is defined as an “unpleasant sensory and emotional experience associated with, or resembling that associated with, actual or potential tissue damage” [32], and its prevention and management have become essential components of high-quality veterinary care [31].

Effective pain management is essential for patients’ physical recovery and overall quality of life, which can adversely affect their well-being, including mobility, appetite, social interactions, and general behavior, as commonly perceived in chronic pain [31,33]. Animals dealing with ongoing cancer issues tend to have a significantly lower quality of life compared to healthy animals, likely due to associated pain [34,35]. Yazbek et al. [36] validated a health-related quality-of-life scale for dogs experiencing cancer pain. So, comprehensive pain management is vital to ensure that oncologic patients can live as comfortably and with as high a quality of life as possible.

Nociception is the physiological process of perceiving pain, which involves the transmission of brain impulses in response to painful stimuli through activating peripheral pain receptors (nociceptors) and their specific sensory nerve fibers (Aδ and C fiber) [37,38]. Distinguishing the different types of pain is important for identifying and assessing pain intensity in companion animals using an established scale that correlates the intensity of the animal’s pain with atypical postures, behavioral changes, postural reactions, pupil diameter, the presence of vocalization, and reactions such as fear and stress [39]. Understanding the neural pathways responsible for processing pain stimuli also allows for a practical analgesic approach based on the patient’s response and condition, promoting adequate pain relief, faster recovery [40], and targeted animal pain-oriented therapy [41].

### 3.1. Concepts of Type Pain, Physiopathology, and Management Approach

“First pain” is the initial response to a painful external stimulus, classified as inflammatory, adaptive, physiological, or acute pain [42]. It is specific to the body’s area, lasts for a short period, and usually results from inflammation or tissue injuries, leading to hypersensitivity in the affected area and promoting an individual’s response to induce tissue repair responses [42]. For managing acute pain or acute exacerbations of chronic pain, multimodal analgesia is considered more effective [40]. Alongside conventional opioid-based analgesia, several drugs with different mechanisms of action exert additive and/or synergistic effects by targeting pain pathways, such as, for example, alpha-2 agonists, N-methyl D-aspartate receptor antagonists (NMDA), dexamethasone, non-steroidal anti-inflammatory drugs (NSAIDs), and acetaminophen in humans [40], dogs and cats [18,43]. 

Nociceptive adaptive pain, also called pathological, clinical, chronic, and “second pain”, arises when the activation of C fibers intensifies the original stimulus, resulting in an intense and enduring pain feeling processed by the central nervous system [44]. It may be localized or triggered by external events, typically lasting longer (between three and six months) or being more severe than usual [44]. This specific type of pain does not provide any beneficial biological function, leading to substantial incapacity and decreasing the patient’s life quality [44]. Unmanaged acute or chronic pain not only causes suffering but also increases vulnerability to health conditions and significantly prolongs recovery times [45,46]. Therefore, a multimodal strategy, such as physiotherapy, pharmacology, surgery, and acupuncture, is essential for effectively controlling this pain, with combined analgesic agents like non-steroidal anti-inflammatory drugs (NSAIDs) remaining the most prevalent therapeutic option [44,47]. Central sensitization is an important issue in both types of pain management. It refers to the increased responsiveness of neurons in the central nervous system to normal or subthreshold afferent input, resulting in pain hypersensitivity [48,49,50,51]. Also, central sensitization frequently results in allodynia (pain from normally non-painful stimuli) and hyperalgesia (increased pain from normally painful stimuli), contributing to chronic pain persistence [48,49,50,51]. Peripheral sensitization occurs when nociceptors in the periphery become more sensitive to inflammatory mediators, leading to an increased response to stimuli [48,49,50,51]. These processes involve complicated interactions between many molecular pathways, such as the activation of NMDA receptors, the upregulation of voltage-gated sodium channels, and the release of cytokines that cause inflammation [48,49,50,51].

Neuropathic pain, or functional non-adaptive pain, arises from damage to the peripheral and/or central somatosensory systems [52]. In this type of pain, it is expected to see a limited response to conventional analgesic drugs such as non-steroidal anti-inflammatory drugs (NSAIDs) and some opioid agents [52]; however, methadone may be a unique opioid that could have a particular benefit in patients with neuropathic pain [53].

Visceral pain, often called non-adaptive neuropathic pain, occurs when visceral nociceptors transmit painful signals through Aδ and C fibers via sympathetic and parasympathetic pathways [54,55]. Most stimuli commonly perceived as painful in soft tissue damage do not cause an equal sensation when applied to the damage in visceral tissue [56]. To improve the visceral pain therapy response, the analgesia must be targeted at the cause of pain and be combined with other analgesic drugs [55].

Somatic pain, also known as central neuropathic non-adaptive pain, can be differentiated as deep somatic pain (that arises by activation of nociceptors in soft tissues, presenting as a general and unlocalized pain) and superficial somatic pain (that originates from the stimulation of nociceptors on the skin surface, presenting a specific and well-defined location) [57]. Although chronic visceral pain and somatic pain have distinct underlying causes, the present approach to managing chronic visceral pain is largely based on the recommendations established for somatic pain [56,57].

### 3.2. Pathophysiology of Pain in the Oncologic Patient

Understanding the neurobiology of pain in oncologic patients necessitates thoroughly examining the processes involved in pain perception, including transduction, transmission, modulation, projection, and perception [58]. Furthermore, the role of central and peripheral sensitization in transforming acute pain into chronic pain is crucial [59]. Transduction is the initial process by which nociceptors turn mechanical, thermal, or chemical pain into electrical signals sent to the spinal cord by peripheral nerves [58]. Afferent pain fibers, notably Aδ and C fibers, are critical in this process [60,61], and tumor growth can interfere with normal nerve function, leading to abnormal signal transmission and enhanced pain responses [62,63]. Moreover, cancer cells and their microenvironment release mediators like prostaglandins E2 (PGE2), tumor necrosis factor-α (TNF-α), endothelins, interleukin-1 and -6, epidermal growth factor, transforming growth factor-β, and platelet-derived growth, that sensitize nociceptors, increasing their responsiveness and contributing to heightened pain sensation [58,61,62,63]. Pain signals, through various neurotransmitters, such as glutamate and receptors, for example, glutamate N-methyl-D-aspartate receptor (NMDA), are modulated primarily in the spinal cord’s dorsal horn and brain centers (including the thalamus and cortex, which are essential for pain localization and intensity), where signals can be amplified or inhibited [64]. The balance between excitatory and inhibitory signals can be disrupted in oncologic patients, altering pain experiences and making the pain more diffused and challenging to manage [65,66].

Oncologic pain is influenced and can result from tumor type (the type of tumor can affect the pain sensitization, changing acute pain into chronic pain through the inflammatory environment created by tumors and direct nerve invasion, perpetuating these sensitization processes) [67,68,69,70] and chemotherapeutic agents (which accumulate in peripheral sensory ganglia and nerves, causing cell death and neuronal degeneration and affecting ion channels such as calcium channels and sodium channels, leading to increased neuronal excitability and pain) [60].

Improved understanding of these mechanisms can lead to novel and targeted approaches for pain relief, moving beyond conventional opioids and standard analgesics. It is believed that effective management of oncologic pain requires a multimodal approach that addresses the neurobiological mechanisms and the individual patient’s needs. This includes using various analgesics, physical therapies, and psychological support to provide comprehensive pain relief.

## 4. Main Opioids Used for Pain Control in Oncological Patients

Opioid agents, despite their side effects, are still the most commonly used analgesic drugs for moderate and severe pain [71], as well for oncologic pain [72]. In human medicine, there are growing concerns about the long-term efficacy and safety of opioids. However, the guidelines still acknowledge this analgesic agent as a viable and effective option for pain management [71]. In veterinary medicine, opioids are also widely used for managing pain in various clinical situations, including cancer [19].

They generally exert their pain-relieving effect by activating inhibitory pathways in the central nervous system (CNS) and pain-sensing neurons in the sympathetic nervous system, reducing pain transmission receptors. There are three main types of opioid receptors: μ (mu), δ (delta), and κ (kappa) receptors. Each of these receptors has a distinct distribution and role in modulating pain [17,73,74].

### 4.1. The Mechanisms of Action of Opioids Rely on the Body’s Receptors

Most opioids used in therapeutic settings primarily target receptors. These structures are highly concentrated in some regions of the brain and spinal cord that are essential for perceiving and regulating pain, such as the periaqueductal grey, rostral ventromedial medulla, and dorsal horn of the spinal cord [15,75]. Activation of β-receptors inhibits adenylate cyclase, leading to a decrease in cyclic AMP (cAMP) levels. This, in turn, causes a reduction in the release of pain-related neurotransmitters such as P and glutamate, which are important for transmitting pain signals [15,75]. Furthermore, the activation of μ-receptors causes neurons to hyperpolarize, increasing their difficulty in generating electrical impulses and conveying pain signals [15,75].

Delta receptors are found in the brain and spinal cord; however, their distribution is more limited compared to μ-receptors [15,75]. These receptors are involved in pain regulation; however, their specific contribution to analgesia is not as well comprehended as μ-receptors [15,75]. They also have an impact on mood control and the emotional component of pain relief [15,75].

Kappa receptors are mostly situated in the central nervous system, namely in the brain and spinal cord, but may also be found in peripheral tissues [15,75]. Stimulation of κ-receptors may provide pain relief, especially in the spinal cord, but it can also lead to feelings of unease and hallucinations, which restricts its use in therapeutic settings [15,75]. Kappa receptors are believed to regulate pain through processes distinct from those of receptors, potentially involving other neurotransmitter systems [15,75].

### 4.2. Morphine, Methadone and Fentanyl as Pure Opioids Agonist

Pure opioid agonists are used to treat moderate and severe pain, as well as to promote anesthesia with combined anesthetic agents [15,76,77]. Throughout analgesia, they also promote many side effects, such as respiratory depression, sedation, nausea, constipation, opioid tolerance, and hyperalgesia [15,76,77].

*Morphine* is a natural opioid alkaloid with a high affinity for µ-receptors, obtained from the *Papaver somniferum poppy* [8,22]. Morphine acts primarily on the activating descending inhibitory pathways of the CNS and inhibition of the nociceptive afferent neurons of the PNS, resulting in an overall reduction of the nociceptive transmission [17]. Its analgesic efficacy and its many routes of administration enhance its practicality in several anesthetic protocols, making morphine one of the opioids more commonly used in clinical practice on oncologic and non-oncologic patients and the analgesic more researched by the scientific community [14,17]. Nevertheless, chronic morphine administration promotes immunosuppression and amendments of immunological indices [78], increasing the infection’s susceptibility [79]. The analgesic efficacy of morphine reduces surgical stress and pain. It is mainly important for cancer human and animal patients, where feeling pain has been linked to an increased risk of tumor spread [3,80,81,82]. Animal studies suggest that morphine provides adequate postoperative analgesia in canines undergoing oncological surgery when compared with tramadol [8], as well as giving a notable antinociceptive effect in cats undergoing ovariohysterectomy under sacrococcygeal epidural with morphine and lidocaine [14]. Research suggests that morphine promotes longer and more potent effects on thermal antinociception induced in cats through the epidural technique, compared with buprenorphine [83], which is the most used opioid agent in epidural administration for dogs and cats [84]. Other animal studies demonstrated that in canines undergoing ovariohysterectomy, there is no significant difference in postoperative analgesic effectiveness between tramadol and morphine [85] as well as with epidural technique with lidocaine/tramadol and lidocaine/morphine [86]. Morphine also provides significant pain relief in a wide range of tumor types, making it a critical component of palliative care [87]. However, morphine analgesia has been associated with promoting tumor cell growth capacity [22] and avoiding morphine or another opioid in managing oncologic pain, an effective alternative strategy for pain control, should be guaranteed [80]. These strategies include regional anesthesia, reduction of opioid dose or opioid-free analgesia, or alternative analgesic interventions [14,25,80,88].

*Methadone* is a synthetic opioid with a highly potent µ-opioid agonist and possesses some affinity for the κ- and δ-opioid receptors [89]. It prevents monoamine reuptake in the periaqueductal grey region of the brain and inhibits presynaptic N-methyl-D-aspartate receptors [89]. The NMDA antagonist properties of methadone could, therefore, make this drug particularly useful for patients resistant to other opioids or with neuropathic pain [53]. Considering its distinct mode of action over other opioids, methadone has gained a particular effect on the treatment of opioid-induced hyperalgesia and central sensitization [90]. In cats and dogs, methadone and buprenorphine are the two most commonly prescribed narcotics for premedication [15]. This is because both medications have a moderate duration of action (methadone lasting 4-5 h and buprenorphine lasting 6–8 h) [15]. As a result, they will effectively provide a significant duration of action for the entire perioperative period for most procedures performed in veterinary practice [15]. In companion animals, methadone was performed to be an enhanced ovariohysterectomy analgesia than butorphanol [91] and buprenorphine [92]. In dogs undergoing ovariohysterectomy, methadone combined with fluconazole provided effective *post-surgical* analgesia [93]. In human and veterinary medicine, methadone has gained increasing attention for managing oncologic [3,53,94,95,96] and noncancer pain [91,97,98], as well as in neuropathic pain [99].

*Fentanyl* is an opioid agonist that acts quickly and has a brief duration of action at the typical clinically administered dosages [15]. The injectable solution is suitable for both dogs and cats as a co-induction agent and for providing analgesia during and after surgery [15,100]. Fentanyl has a rapid onset of action, taking less than 5 min when given as a bolus [15]. This makes it very effective for providing pain relief in response to surgical stimulation during surgery [15]. If the surgical stimulation persists, we can provide a continuous intravenous infusion and adjust the rate of administration to achieve the desired effect [15]. Additional doses can be administered as necessary [15]. When a patient is under general anesthesia, giving them a fentanyl bolus or infusion might result in bradycardia, respiratory depression, or even apnea [15]. We recommend stopping the fentanyl infusion at least 15 min before the end of anesthesia to prevent respiratory depression during the recovery phase [15]. The occurrence of respiratory depression in aware individuals is quite improbable [15]. In dogs and cats, fentanyl has been extensively used via fentanyl patches for long-term pain relief, despite their lack of official approval [101,102]. Remifentanil, due to its potency and short duration of action, remains an unofficially approved opioid drug [15]. It targets mu-opioid receptors in the body [15]. This substance’s onset and duration of action are shorter than 6 s, indicating that it is only appropriate for intravenous infusion [15]. A bolus does not need to be administered and the dosage rate can be quickly adjusted [15]. The short duration of action results from esterase enzymes’ breakdown in the bloodstream [15]. The primary use is to provide pain relief during surgery for individuals with impaired liver function [15]. It is crucial to provide analgesia with an alternative opioid throughout the postoperative period, as its effects quickly resolve upon discontinuation [15]. In dogs, in the study conditions, remifentanil efficacy in reducing sevoflurane minimum alveolar concentration did not diminish in the short term, suggesting remifentanil did not induce acute opioid tolerance [76]. Hyperalgesia was not detected 3 or 7 days after remifentanil administration [76]. However, the development of acute opioid tolerance or opioid-induced hyperalgesia in dogs is not supported by the findings of this study [76].

### 4.3. Buprenorphine, a Partial Opioid Agonist

Buprenorphine has a more prolonged onset of action, which makes it less suitable for intraoperative administration to enhance analgesia [15]. Postoperative opioids are chosen based on the expected degree of pain after surgery, using buprenorphine for patients with light or moderate pain if a multimodal analgesia strategy is in place [15]. Recent evidence indicates that bitches who receive methadone as premedication before undergoing ovariohysterectomy experience lower pain scores for the first 8 h after surgery compared to those who receive buprenorphine [15]. This supports the use of methadone as the preferred premedication for dogs undergoing ovariohysterectomy [92]. Research has shown that buprenorphine offers long-lasting pain relief while causing minimal adverse effects [83]. As a result, it is considered a favorable choice for treating moderate to severe pain in cats and dogs [83].

### 4.4. Butorphanol, an Opioid Agonist-Antagonist

Butorphanol is a pharmacological chemical that operates as a dual-acting drug, acting as both an agonist and antagonist, with a special focus on the kappa receptors [15]. Compared to other opioids, its effectiveness is limited [15]. It offers less effective pain relief compared to methadone and buprenorphine, which is why it is not advised for use as a premedicant before most surgical operations [15]. Compelling data indicates that butorphanol is not effective in providing sufficient pain relief for cats undergoing ovariohysterectomy, especially when they are premedicated with acepromazine. Butorphanol, functioning as a depressant of the cough center, has been authorized as an antitussive medication. Additionally, it may be beneficial to counteract the effects at one receptor type while still producing an impact at a different receptor type [15].

### 4.5. Tramadol, an Atypical Opioid

Tramadol hydrochloride, an atypical opioid, is a codeine-derived synthetic analgesic prescribed for the management of moderate-to-severe pain [17]. It operates within the central nervous system by inhibiting neuronal reuptake of norepinephrine and serotonin and by interacting synergistically through two mechanisms: μ-opioid receptor agonist and inhibition of neuronal reuptake of norepinephrine and serotonin [103,104]. It binds to μ-opioid receptors with minimal affinity [17,105]. While tramadol provides adequate postoperative analgesia compared to morphine in canines undergoing oncological surgery [8], some studies have shown it to be less effective overall [13]. According to the available data, tramadol seems to be a more suitable therapeutic option for cats [106] and should preferably be used as a component of multimodal analgesia in both species [105]. In a unilateral canine mastectomy, tramadol in combination with meloxicam presents less analgesic efficacy in comparison to opioids, namely morphine [107]. In surgical excision of cutaneous tumors in canines, no significant difference in postoperative pain relief was detected between pre-emptive administration of carprofen and tramadol or no pre-emptive pain relief [108]. Administration of tramadol with NSAID can enhance the analgesic efficacy and promote relief of moderate to severe pain [3].

The recommended dose, frequency, and route of administration for the most common opioid analgesic drugs used in dogs and cats is described in Table 1, according to the WSAVA Global Pain Management Guidelines [18].

## 5. The “Conflicting Relationship” between Opioids and the Immune System

Researchers have linked opioid analgesic agents to a detrimental impact on the number and function of immune cells [109]. Opioids modulate the immune system response via the endocrine and nervous systems [110] and opioid receptors on the surface of immune cells [109]. They promote systemic adverse effects, including inhibiting cellular and humoral immune function [10] and increasing infection susceptibility [111].

In animal studies, morphine’s high affinity for µ-receptors is proposed as a primary mechanism for regulating humoral and cellular responses by reducing macrophage and natural killer cell activity [109,112], decreasing migration and function of leukocytes during the initial innate phase, suppressing Natural Killer (NK) cell activity [22] and cytotoxicity [110], and altering the immune and apoptotic pathways in canine leukocytes [113]. They can also alter phagocytic function, cytokine production, and leukocyte apoptosis [114] and have direct effects through opioid receptors expressed on macrophages, neutrophils, and lymphocytes [115]. For example, they suppress splenic macrophage function, cytokine production, and costimulatory molecules [116]. Additionally, high and low morphine doses can influence the natural killer cell’s cytotoxicity differently [117,118]. High-dose morphine in mice impaired angiogenesis, increased systemic oxidative stress and decreased the mobilization of endothelial progenitor cells [119]. It does not affect healthy dogs’ leukocyte cytokine production or neutrophil phagocytic activity [120]. Similar findings from studies on humans suggest that morphine stops macrophages from phagocytosis and stops the production of interleukin-2 and γ-interferon [10]. Morphine can shift lymphocytes T-helper 1 to T-helper 2, promoting an imbalance in the Th1/Th2 ratio [121] and increasing the risk of morphine-treated individuals getting infections [111]. However, a few studies suggest that chronic administration of morphine in humans shows beneficial results in a significant decrease in inflammation-induced angiogenesis, a substantial reduction in the expression and nuclear translocation of HIF-1 alpha with a concurrent suppression in vascular endothelial growth factor (VEGF) synthesis, and inhibited early recruitment of both neutrophils and monocytes towards an inflammatory signal with a significant decrease in the monocyte chemoattractant MCP-1 [122]. Studies have demonstrated that low daily doses of morphine might promote tumor cell proliferation, angiogenesis, and immunosuppression [22,123].

Tramadol is a commonly used opioid analgesic in dogs, particularly those with compromised immune systems [124]. Their pharmacological behavior may explain their different impact on the immune system compared to other opioids, such as non-suppression of cellular immunity functions, increased activity of NK cells, proliferation of lymphocytes, and production of interleukin-2 [125]. Regardless of the tramadol dose in dogs, animal studies found that it did not alter the leukocytes’ production of cytokines, making it a good analgesic for animals with immune impairment and infection risk [124]. In dogs, studies suggest that tramadol has a minimal effect on phagocytosis and oxidative burst and may promote a proinflammatory shift [124]. Human studies also found that tramadol had a low impact on humoral and cellular immunity, suggesting it is a choice for post-surgical pain control [22]. They also indicated that tramadol can shift T helper cells to Th2, promoting an imbalance in the Th1/Th2 ratio that is less potent than morphine [121]. Although tramadol can inhibit the proliferation of lymphocytes, it can also increase the NK’s activity [22].

Methadone is a commonly used analgesic for both cancer and noncancer patients, but its impact on the immune system remains uncertain due to a shortage of scientific research. In animal studies, methadone prenatal exposure in rats alters peripheral inflammatory and central immune characteristics and causes immune hyperreactivity [126]. In human studies, methadone has been demonstrated to have a minimal effect on both humoral and cellular immunity [127], but chronic administration presents a restraint on immunocyte count and activity [128].

Fentanyl induces dose-related immunosuppression similar to morphine. In rodents, continuous fentanyl infusion suppresses natural killer (NK) cell activity, lymphocyte proliferation, and cytokine production. Studies using tumor cell lines injected into rodents showed that fentanyl-induced suppression of NK cells led to an increase in lung metastases [129]. Fentanyl also exacerbates surgery-induced immunosuppression, but chronic administration may lead to tolerance of its immunosuppressive effects. Fentanyl’s immunosuppressive properties are also well-documented in clinical settings. During the perioperative period, fentanyl suppresses NK cell cytotoxicity and reduces proinflammatory cytokines such as IL-1 and IL-6 [130,131].

Butorphanol has been noted to affect the activity of immune cells such as macrophages. In both in vitro and in vivo studies with mice, butorphanol significantly shifted macrophages to the M2 phenotype. It greatly reduced the expression of IL-6, TNF-α, and iNOS in LPS-stimulated bone marrow-derived macrophages [132].

Buprenorphine can modulate T cell function, reduce T cell proliferation and cytokine production, and reduce the production of proinflammatory cytokines, similar to other opioids [120]. Some authors say that in humans, buprenorphine demonstrates a neutral behavior on the immune system at the doses used for analgesia [131,133].

The dose-related effects of fentanyl, butorphanol, buprenorphine, methadone, tramadol, and morphine on the immune system are described in Table 2.

## 6. The Opioid Agents as Influencers of Tumor Survival

Opioid agents remain the primary method of pain management in oncology patients, despite numerous studies suggesting their association with tumor growth [22] by the presence of opioid receptors in cancer cells [137] and by suppression of the immune system response [21,22]. According to animal studies, this factor could decrease the average animal’s lifespan with oncologic conditions [138,139]. To mitigate the side effects of opioid agents, it is advisable to consider and explore anesthesia with low-opioids or opioid-free anesthesia as a new possible alternative for immune-suppressing and oncologic patients [140]. Conversely, studies propose that the analgesic ability of opioids can mitigate the impact of pain-inducing stressor factors, such as decreases in the number and function of immune cells [25].

Several animal studies suggest that morphine influences chemoresistance by boosting the number of cancer stem cells [141], promoting tumor development [141,142], and stimulating tumor angiogenesis [143]. However, additional research on animals has demonstrated that morphine can diminish the spread of cancer cells to other parts of the organism [144], inhibit tumor growth and spread [145], mitigate the spread of cancer cells caused by surgery [146], and prevent the formation of new blood vessels in cells by decreasing their oxygen supplies [147]. This potential dual role of morphine on cancer progression is influenced by both the dosage of morphine and specific tumor type. The mechanism behind the dose-dependent effects of morphine is not yet fully understood. It is suggested that high concentrations of morphine are believed to suppress tumor cell growth and inhibit angiogenesis and metastasis.

In contrast, low daily doses might promote tumor cell proliferation, angiogenesis, and immunosuppression [123]. Human studies demonstrate that morphine has been found to enhance cell migration in breast cancer cells [148] while simultaneously reducing the movement of tumor-infiltrating leukocytes and preventing the formation of new blood vessels that support tumor growth [149]. However, several human investigations have proposed that morphine exhibits anticancer properties, suggesting that morphine can effectively inhibit the growth of lung [147], breast tumors [144], and melanoma [145].

In a preclinical model of ovarian carcinoma in female CB17 SCID mice, the findings indicate that the use of buprenorphine for acute, perioperative pain management did not have any effect on tumor progression [150] or primary tumor growth in mouse surgery osteosarcoma, which is not influenced by pain management with buprenorphine and meloxicam [151].

Human tumor studies reported the effect of butorphanol on cancer cells, which demonstrated its capacity to inhibit angiogenesis and migration and protect PC12 cells against oxygen-glucose deprivation/reoxygenation-induced inflammation and apoptosis [135].

Research on the effects of fentanyl on natural killer cell activity and resistance to tumor metastasis in rats shows that fentanyl suppresses natural killer cells and increases the risk of tumor metastasis [134].

Tramadol has demonstrated anti-tumor activity in animal studies by inhibiting and reducing postoperative recurrence, improving survival [152], and preventing metastatic colonization [153]. In human studies, tramadol as a rescue analgesic after breast cancer surgery reduced the recurrence of cancer and mortality [154]. It has cytotoxic effects in breast cancer cells at concentrations higher than 0.5 mg/mL, inhibiting tumor progression [155].

Recently, methadone has gained much attention as a potential antineoplastic compound because of its apoptosis capacity in cancerous cells or tissues [152,153]. Other studies have found that methadone can promote tumor development [154]. Furthermore, a few human and animal studies are available, and the effect of methadone on tumor growth and spread still needs to be defined.

**Figure 1:**
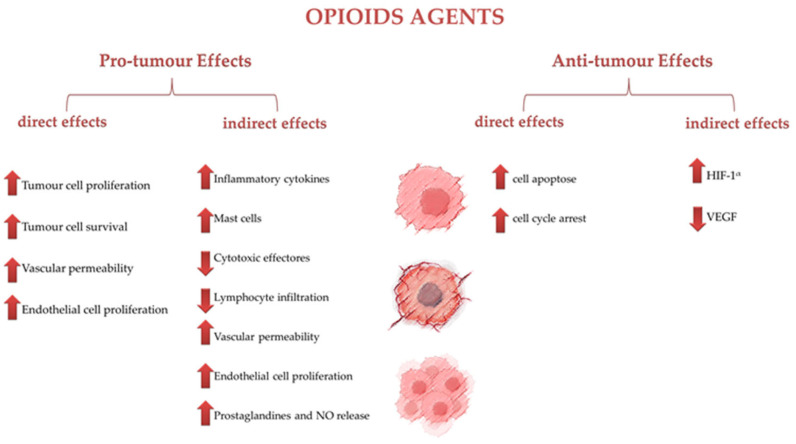
Effects of opioid agents on tumor cells.

Figure 1 resumes the effects of opioid agents on tumor cells, categorized into pro-tumor and antitumor effects, both direct and indirect.

## 7. Limitations of Opioid Use for Cancer Pain Management in Dogs and Cats and Further Research

The use of opioids for managing cancer-related pain in dogs and cats requires careful consideration of factors such as limited availability, legal obligations, potential tolerance development, swallowing difficulties, and taste issues [18].

Regulatory limitations and supply challenges can limit the availability of opioids for veterinary use, and adherence to prescription regimens may be difficult [156]. Veterinarians administer opioids at clinics to create a controlled environment, but this practice can lead to non-compliance, inappropriate doses, and a lack of expert supervision [156]. Additionally, pet owners may abuse opioids and redirect them for human consumption, emphasizing the need for careful prescription and supervision [156].

Oral opioid administration is complex for cats due to first-pass metabolism and palatability concerns [156]. Tolerance to opioids is a concern, but it may not be a significant problem for many animals due to their shorter lifespans [157]. However, developing tolerance requires larger dosages to produce the same pain-relieving effect, increasing the likelihood of adverse effects [157]. Administering different opioids at different points of the perioperative period can enhance pain management, decrease the possibility of developing tolerance, and limit adverse effects by targeting distinct pain pathways during therapy [4]. Premedications can provide early analgesia and sedation, while alternative opioids can be administered during anesthesia and recovery. However, studies have not sufficiently examined the impact and effectiveness of opioids in treating cancer-related pain in many animal species, particularly dogs and cats. Future research should focus on species-specific investigations to better understand the impact of different forms of cancer on dogs and cats, as well as their responses to various opiate therapies [4].

There is a lack of extensive research specifically focused on the effects and efficacy of opioids in managing cancer pain in different species, particularly in dogs and cats. Most existing studies are extrapolated from human medicine or general veterinary practice, which may not account for species-specific opioid responses. Moreover, accurate assessment of pain in veterinary patients remains challenging. Subjective pain scales and behavioral assessments can vary widely between practitioners, leading to inconsistencies in treatment efficacy evaluations. While opioids are effective for short-term pain relief, there is limited data on the long-term effects and safety of chronic opioid use in veterinary oncology patients. Potential issues such as tolerance, dependence, and immunosuppression are areas of concern that need further investigation. Future research should focus on conducting species-specific studies to understand better how different types of cancer affect dogs and cats and how these species respond to various opioid treatments. This includes pharmacokinetics, pharmacodynamics, and optimal dosing regimens for different cancers. Exploring multimodal pain management strategies that combine opioids with other analgesics and non-pharmacological interventions could provide better pain control while minimizing opioid-related side effects. Research should also investigate the efficacy of these combinations in different types of cancer commonly seen in dogs and cats, such as lymphoma, osteosarcoma, and mammary tumors.

## 8. Conclusions

Opioid agents have long been used to treat moderate and severe pain in both oncologic and non-oncologic conditions. However, several studies demonstrated the presence of many side effects of most opioids, such as an imbalance of vital signs, increased predisposition to infection, drug dependence, immune suppression, and influencing tumor growth capacity.

Morphine, until recently, was considered the most widely used agent in human and veterinary medicine for painful disorders despite research demonstrating that it increases tumor cell proliferation and suppresses immune responses. Methadone is an effective analgesic, but its effects on the immune system and tumor cells remain unknown due to insufficient scientific research. While tramadol offers pro-immune and antitumor benefits, its lower analgesic potency necessitates supplementary analgesia.

To mitigate the side effects of opioids, multimodal pain management and opioid-sparing strategies, such as low-dose opioid or opioid-free anesthesia, are recommended. These approaches require the integration of various analgesic agents to ensure effective pain control, given that complete opioid avoidance may not be feasible in many oncologic cases.

Further research is needed to explore the pro-immune and antitumor properties of specific opioids to enhance pain management while minimizing tumor risks.

## Figures and Tables

**Table 1:** Recommended dose, frequency, and route of administration for the most common opioid analgesic drugs used in dogs and cats [18].

Opioid Analgesic	Dogs		Cats		Route of Administration ^2^	Main Indications for Use?
	Dose ^2^	Frequency ^2^	Dose ^2^	Frequency ^2^		
Morphine	0.3 to 0.5 mg/kg	every 2 to 4 h	0.2 to 0.3 mg/kg	every 4 to 6 h	IM, IV *	Moderate-to-severe pain ‡
Methadone	0.3 to 0.5 mg/kg	every 3 to 4 h	0.2 to 0.3 mg/kg	every 4 h	IM, IV, oral transmucosal †	Moderate-to-severe pain
Fentanyl	2 to 5 μg/kg 3 to 6 μg/kg/h	Bolus CRI	1 to 3 μg/kg 2 to 3 μg/kg/h	Bolus CRI	IV	Severe pain
Remifentanil	6 to 12 μg/kg/h	CRI	4 to 6 μg/kg/h	CRI	IV	Severe pain
Buprenorphine	0.01 to 0.02 mg/kg	every 4 to 8 h	0.02 to 0.04 mg/kg	every 4 to 8 h	IM, IV, oral transmucosal †	Moderate pain
Butorphanol	0.2 to 0.4 mg/kg	every 1 to 2 h	0.2 to 0.4 mg/kg	every 1 to 2 h	IM, IV	Mild pain
Tramadol	NR ^¥^	NR ^¥^	2 to 4 mg/kg	every 8 to 12 h	Orally, IM, IV	Chronic pain

Legend: CRI, continuous rate infusion; NR, not recommended. ^2^ Based on individual response and other concomitant treatments. * Only if slow and after drug dilution and still with a risk of histamine release. † Only in cats. ‡ Ideally avoided due to side effects. IM, intramuscular. IV, intravenous. H, hour. ^¥^ Not recommended due to low plasma concentrations of the opioid metabolite O-dimethyl-tramadol; M1, metabolite.

**Table 2:** Dose-related effects of fentanyl, butorphanol, buprenorphine, methadone, tramadol, and morphine on the immune system.

Drug	Dose	Effect on Immune System	Study Model	References
Fentanyl	Low (1–5 μg/kg) and High (75–100 μg/kg)	Suppression of NK cell activity	Human (perioperative)	[134]
	Chronic administration	Suppression of NK activity, lymphocyte proliferation, and cytokine production	Rodent	[129]
Butorphanol	Low	Enhanced macrophage phagocytic activity; balanced cytokine production	Rodent	[135]
	Moderate	Reduced IL-6 and TNF-α	Rodent	[132]
	High	Increased immunosuppressive effects; potential for reduced inflammatory responses	Rodent	[132]
Buprenorphine	Low to Moderate	Modulation of T cell function; reduced proinflammatory cytokine production	Rodent	[120]
	Chronic administration	Development of tolerance to immunosuppressive effects	Rodent	[129]
Methadone	Low to Moderate	Suppresses lymphocyte proliferation; reduces cytokine production	Rodent	[136]
Tramadol	Low to Moderate	Minimal immunosuppressive effects; can enhance certain immune functions	Rodent	[125]
Morphine	Low	Suppresses NK cell activity and cytokine production; increases susceptibility to infections	Rodent	[136]
	High	Significant immunosuppression; decreased macrophage function and cytokine production	Rodent	[136]
	Chronic administration	Development of tolerance to immunosuppressive effects; persistent analgesic effects	Rodent	[136]
